# Associations between the human intestinal microbiota, *Lactobacillus rhamnosus* GG and serum lipids indicated by integrated analysis of high-throughput profiling data

**DOI:** 10.7717/peerj.32

**Published:** 2013-02-26

**Authors:** Leo Lahti, Anne Salonen, Riina A. Kekkonen, Jarkko Salojärvi, Jonna Jalanka-Tuovinen, Airi Palva, Matej Orešič, Willem M. de Vos

**Affiliations:** 1 Department of Veterinary Biosciences, University of Helsinki, Finland; 2 Laboratory of Microbiology, Wageningen University, Wageningen, Netherlands; 3 Department of Bacteriology and Immunology, Haartman Institute, University of Helsinki, Finland; 4 Valio R&D, Helsinki, Finland; 5 Quantitative Biology and Bioinformatics, VTT Technical Research Centre of Finland, Espoo, Finland

**Keywords:** Microbiota, Lipidomics, Probiotics, *Lactobacillus rhamnosus* GG, Gastrointestinal tract, Physiology, High-throughput profiling

## Abstract

Accumulating evidence indicates that the intestinal microbiota regulates our physiology and metabolism. Bacteria marketed as probiotics confer health benefits that may arise from their ability to affect the microbiota. Here high-throughput screening of the intestinal microbiota was carried out and integrated with serum lipidomic profiling data to study the impact of probiotic intervention on the intestinal ecosystem, and to explore the associations between the intestinal bacteria and serum lipids. We performed a comprehensive intestinal microbiota analysis using a phylogenetic microarray before and after *Lactobacillus rhamnosus* GG intervention. While a specific increase in the *L. rhamnosus*-related bacteria was observed during the intervention, no other changes in the composition or stability of the microbiota were detected. After the intervention, lactobacilli returned to their initial levels. As previously reported, also the serum lipid profiles remained unaltered during the intervention. Based on a high-resolution microbiota analysis, intake of *L. rhamnosus* GG did not modify the composition of the intestinal ecosystem in healthy adults, indicating that probiotics confer their health effects by other mechanisms. The most prevailing association between the gut microbiota and lipid profiles was a strong positive correlation between uncultured phylotypes of *Ruminococcus gnavus*-group and polyunsaturated serum triglycerides of dietary origin. Moreover, a positive correlation was detected between serum cholesterol and *Collinsella (Coriobacteriaceae)*. These associations identified with the spectrometric lipidome profiling were corroborated by enzymatically determined cholesterol and triglyceride levels. *Actinomycetaceae* correlated negatively with triglycerides of highly unsaturated fatty acids while a set of Proteobacteria showed negative correlation with ether phosphatidylcholines. Our results suggest that several members of the Firmicutes, Actinobacteria and Proteobacteria may be involved in the metabolism of dietary and endogenous lipids, and provide a scientific rationale for further human studies to explore the role of intestinal microbes in host lipid metabolism.

## Introduction

Over 90% of the cells in the human body constitute microbes, the majority of them living in the lower part of the gastrointestinal (GI) tract. Hence, we are composite organisms programmed not only by the inherited and stable human genome but also by the environmentally acquired and plastic microbiome. The coding capacity of the microbiome vastly surpasses the human genome with more than three million genes (Qin et al., 2010). While many of the microbial functions have not yet been characterized, various mechanisms have been described by which the intestinal microbes impact our life, including the metabolism of our food, exclusion of pathogens and many signaling functions that range from modulation of the mucosal immune response to development of metabolic diseases (Holmes et al., 2011; Sekirov, 2010). Bacterial metabolism of dietary and endogenous substances is known to generate a wide repertoire of metabolites that may have beneficial or harmful effects on the host (O’Keefe, 2008; Blaut & Clavel, 2007). Hence, the intestinal microbiota has a remarkable potential to influence the physiology and biology of the host (Tremaroli & Bäckhed, 2012), and unlike the human genome, its gene pool can be modulated by changing the environmental conditions, such as food or drug intake, affecting the composition and function of the intestinal microbiota (Zoetendal, Rajilić-Stojanović & de Vos, 2008).

Consumption of lactic acid bacteria marketed as probiotics is a common approach to maintain health (Saxelin et al., 2005). *Lactobacillus rhamnosus* GG is one of the most widely used probiotic bacteria that is assumed to interact with the host via binding to human mucus via its extracellular pili (Kankainen et al., 2009). However, the further molecular details of the probiotic signaling are not yet understood and it remains to be established whether the effect is direct, through metabolites or structural components modulating for instance the immune responses of the host or indirect, via alteration of the intestinal microbiota. From the ecological perspective, a single bacterial strain is not likely to radically alter the established intestinal community, which in adults typically consists of hundreds of different species-level phylotypes that represent approximately ten bacterial phyla, vastly dominated by Firmicutes, Bacteroidetes and Actinobacteria, followed by Proteobacteria, Fusobacteria and Verrucomicrobia (Zoetendal, Rajilić-Stojanović & de Vos, 2008). Many of the currently characterized phylotypes are strict anaerobes, have not yet been cultured, and can only be recognized based on molecular methods (Zoetendal, Rajilić-Stojanović & de Vos, 2008). The development of culture-independent molecular techniques has provided insights in the composition of the intestinal microbiota before and following probiotic intake. Targeted microbiota analyses as well as the more recent community-level profiling studies support the view that the effects of probiotic intake are limited and only affect bacteria related to the probiotics (Satokari et al., 2001; Vaughan, Mollet & de Vos, 1999; Palaria, Johnson-Kanda & O’Sullivan, 2011; McNulty et al., 2011).

Animal and *in vitro* studies have shown that the intestinal microbiota can regulate host lipid metabolism via numerous microbial activities (Fava, 2006; Martin et al., 2007). The best characterized mechanism is through the biotransformation of bile acids, which regulates the digestion and absorption of fats, and profoundly affects the cholesterol and other lipid metabolism in the body (Gérard, 2010; Ridlon, Kang & Hylemon, 2006). However, global monitoring of the serum and organ lipid profiles of germ-free and conventionally raised mice suggests an even more widespread and profound influence of the intestinal microbiota on host lipid metabolism, in particular on triglycerides and phosphatidylcholines (Velagapudi et al., 2010; Orešič, Hänninen, & Vidal-Puig, 2008).

In the present study, we characterized the impact of a probiotic intervention on the composition and stability of the intestinal microbiota and serum lipid profiles in healthy adults. A comprehensive intestinal microbiota profiling and phylogenetic analysis was carried out with the Human Intestinal Tract Chip (HITChip), a phylogenetic microarray that provides a robust and sensitive measurement platform to assessing the abundance of over 1000 microbial species-like phylotypes representing the majority of the known bacterial diversity of the human intestinal tract (Rajilić-Stojanović et al., 2009). Stability of the microbiota was quantified by inter- and intra-individual correlations within and between time points. The study subjects were sampled prior and after three weeks consumption of dairy milks supplemented with either the probiotic *L. rhamnosus* GG or a placebo. Moreover, the microbiota composition was analyzed from follow-up samples collected three weeks after the intervention trial. With phylogenetic microarray analysis we could both deepen and expand the typical bacterial analysis in probiotic trials, often limited to specific bacterial groups, such as lactic acid bacteria, or to the dominant fraction of the microbiota. Previously, we have shown that the intake of *L. rhamnosus* GG did not elicit any significant changes in the serum lipids (Kekkonen et al., 2008b). The present study complements these results by providing longitudinal data and evidence that the probiotic intervention with *L. rhamnosus* GG does not affect the overall composition or stability of the intestinal microbiota. Furthermore, we have integrated the lipid profiling and global microbiota data sets to investigate the overall associations between the intestinal microbes and systemic metabolites, in particular serum lipids. Quantitative analyses suggest that the abundance of specific intestinal microbes is correlated to that of specific lipid species. We identified mainly uncultured members of the Firmicutes, Actinobacteria and Proteobacteria that seem to be involved in the absorption and metabolism of the dietary and endogenous lipids, providing a scientific rationale for further human studies designed to explore the role of intestinal microbes in lipid metabolism, and associations to health.

## Materials and Methods

### Ethics statement

The trial and its protocol have been approved by the Ethics Committee of the Hospital District of Helsinki and Uusimaa (Ethical protocol no HUS 357/E0/05). The subjects provided written informed consent. The details of this trial have been published previously (Kekkonen et al., 2008a; Kekkonen et al., 2008b).

### Subjects

**Figure 1:**
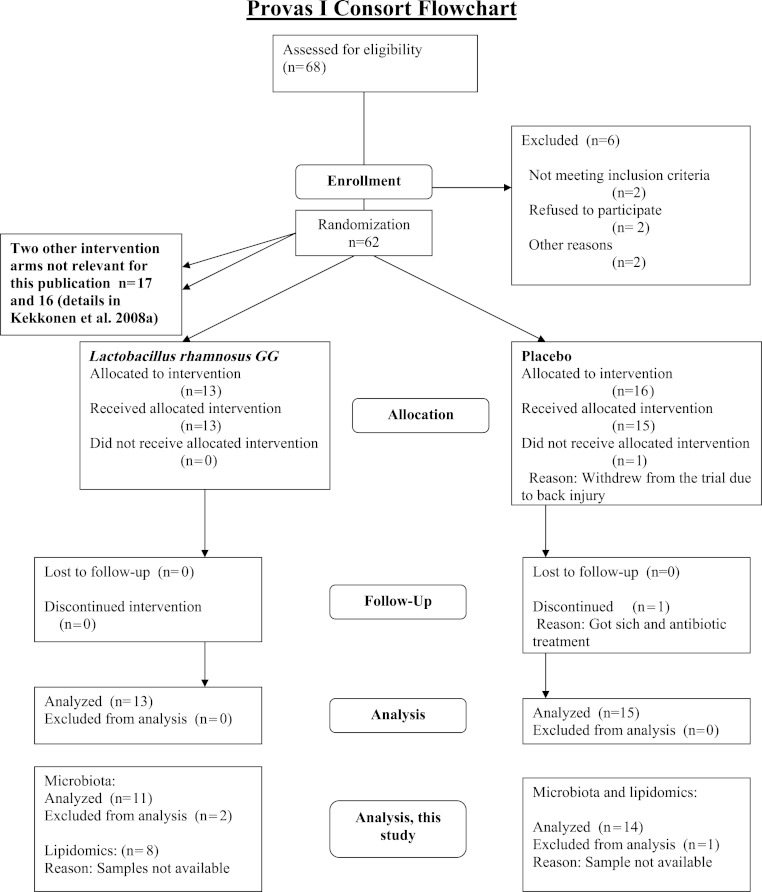
Flowchart of the study subjects during the trial.

The subjects were healthy Finnish adults (*n* = 25, 18 females, 7 males) with a mean age of 42 years (23–55) and a mean BMI of 24 kg/m^2^ (18–30) from Helsinki region, representing a subset of a larger cohort (Kekkonen et al., 2008a; Kekkonen et al., 2008b). The CONSORT flowchart (Fig. 1) summarizes the subject enrollment, intervention allocation, follow-up, and data analysis during the study. The subject characteristics are provided in Supplemental Table S1.

### Dietary intervention

The subjects constituted two treatment groups in a randomized, double blind intervention study to receive either *L. rhamnosus* GG (probiotic; *n* = 11) or placebo (*n* = 14). During the intervention the subjects consumed daily a 250 mL milk-based fruit drink containing either *L. rhamnosus* GG (ATCC 53103, 6.2 × 10^7^ cfu/mL) or a similar placebo drink without probiotic bacteria for three weeks. No other probiotic-containing products were allowed three weeks prior or during the intervention; a list of fermented foods and commercial probiotic-containing products was given to the subjects. The subjects were not separately questioned about their abstinence from probiotic products, but they filled a study diary recording the daily intake of the study product.

### Fecal samples and microbiota analysis with the HITChip and quantitative PCR

Three fecal samples per individual were collected three weeks before, during and three weeks after the intervention, as previously described (Kekkonen et al., 2008b). The fecal DNA extraction with modified Promega method, and quantification of the probiotic counts with a strain-specific quantitative PCR (qPCR) assay have been described previously (Ahlroos & Tynkkynen, 2009). The phylogenetic analysis of the intestinal microbiota composition with the HITChip microarray was performed as previously described (Rajilić-Stojanović et al., 2009; Salonen et al., 2010). The phylogenetic HITChip microarray targets the V1 and V6 hypervariable regions of the 16S rRNA gene of over 1000 bacterial phylotypes that present the majority of the so far detected phylotypes of the human intestine. Phylogenetic organization of the microarray probes and data preprocessing have been explained in detail elsewhere (Rajilić-Stojanović et al., 2009; Salonen et al., 2010; Jalanka-Tuovinen et al., 2011). Quantification of the relative differences in the taxon abundance between samples was obtained by summarizing the probe signals to phylotype (species-like), genus and phylum levels. Genus-level taxa with ≥ 90% sequence similarity in the 16S rRNA gene are referred to as *Species* and relatives, the latter being shortened in the text as “*et rel*.” (Rajilić-Stojanović et al., 2009).

Previously, HITChip-derived microbial profiles have been shown to correlate well with those obtained with fluorescence in situ hybridization (FISH) (Rajilić-Stojanović et al., 2009) and pyrosequencing (Claesson et al., 2009). Samples were also used for absolute quantification of total bacteria, methanogenic Archaea, *Lactobacillus* group and *Bifidobacterium* spp. with previously described qPCR primers and reaction conditions (Salonen et al., 2010).

### Blood samples and their biochemical analyses

Of the 25 study subjects used for the microbiota analysis, 22 (8 from probiotic group and 14 from placebo group) were available for the parallel analysis of serum lipid profiles before and after the intervention (44 samples in total). Venous blood samples from the antecubital vein were taken at baseline and after the three-week intervention. The blood samples were stored at −20 °C for further analyses. Total cholesterol, high-density lipoprotein (HDL) and low-density lipoprotein (LDL) cholesterol as well as triglyceride levels were enzymatically determined from the serum as previously described (Kekkonen et al., 2008a).

### Global lipid profiling: sample preparation and analysis by UPLC-MS

Extraction of lipids from the serum samples, analysis of the lipid extracts with Waters Q-Tof Premier mass spectrometer combined with an Acquity Ultra Performance LC^TM^ (UPLC), data preprocessing and identification of the lipid molecular species is described in detail elsewhere (Kekkonen et al., 2008a). Lipids have been named according to Lipid Maps (http://www.lipidmaps.org) with the following abbreviations: Cer: ceramide; ChoE: cholesteryl ester; lysoPC: lysophosphatidylcholine; PA: phosphatidic acid; PG: phosphatidylglycerol; PC: phosphatidylcholine; PS: phosphatidylserine; SM: sphingomyelin; TG: triglyceride. Where the fatty acid composition could not be determined, the total number of carbons and double bonds is indicated. The first number indicates the amount of carbon atoms in the fatty acid molecule, followed by the number of double bonds.

### Statistical analyses

The effects of probiotic intervention on the intestinal microbiota were investigated with a combination of linear models, sparse principal component analysis (PCA; Shen & Huang, 2008; Lê Cao, González & Dèjean, 2009), unsupervised hierarchical clustering, and significance testing. The log-transformed HITChip and lipid profiling values were approximately Gaussian distributed and fulfilled the general statistical assumptions underlying the selected computational approaches. Background variables, including age, body-mass index and gender were compared in the baseline samples to exclude potentially confounding effects associated with these variables. The Wilcoxon test was used with continuous variables and the Fisher exact test for categorical variables, followed by Benjamini–Hochberg *p*-value correction for multiple testing. No significant differences between the background variables were observed between the treatment groups (*p* > 0.05 in all comparisons). The two-group comparisons between the time points and between the treatment groups were quantified based on a linear model with group-wise fixed effects and sample-specific random effects, as implemented in the limma R package (Smyth, 2004), to identify bacterial taxa with significant changes induced by the probiotic intervention and to assess the magnitude and significance of the effects. The function lmFit was used to fit the linear model, followed by significance estimation by empirical Bayes as described in Smyth (2004). In addition, power calculation was carried out to assess statistical power of the current study. The original data was randomly permuted, one significant 2-fold alteration was inserted, and Gaussian noise was added using the same average standard deviation as in the original data. Empirical power calculation with 1000 random permutations showed that 2-fold and higher alterations that follow the noise levels in the original data were detected in > 99.8% of the cases with sample size of 8 or more, based on the same detection criteria than in the current study, confirming the high statistical power of the analysis at the present sample size. PCA is a linear dimension reduction method used to compress information in the high-dimensional phylogenetic (genus-level) and lipid profiles into few informative features that capture the main variation in the data and allow two-dimensional visualization of sample similarities. The temporal and inter-individual similarity of the microbiota and lipid profiles was assessed by average intra- and inter-individual Pearson correlations (*r*) between and within the time points, respectively. The differences in profile similarity between the probiotic and placebo groups were estimated with Wilcoxon test. The biweight midcorrelation, which is more robust to outliers than the standard Pearson correlation, was used to quantify correlation between the microbiota and lipid profiles across the individuals. High-throughput screening studies involve considerable multiple testing and the traditional multiple testing correction approaches are prohibitively conservative in this context due to their emphasis on estimating the probability of a single false positive finding. Hence, we have used *q*-values for multiple testing correction in the high-throughput screening tests that include parallel comparisons of large numbers of lipids and bacterial phylotypes.

### Correlation analysis of the intestinal microbiota and serum lipids

The biweight midcorrelation measure was used to quantify associations between the microbiota and lipid profiling data sets, and between the microbiota and biochemically determined lipids. The correlations between genus-level bacterial groups and lipid species were calculated together with significance estimates that were corrected for multiple testing. The significantly correlated (*q* < 0.05) phylotypes and lipids were selected for further analysis. It is important to note, however, that while the present sample size of 22 samples across 2 time points (before and during the probiotic intervention) is sufficient for highlighting significant correlation patterns, the present experimental design cannot uncover causal relationships and the observed correlations may be partly associated with diet or other confounding variables that simultaneously affect both lipid and microbiota profiles. Two-way average linkage hierarchical clustering of the bacterial taxa and lipids was applied to highlight and visualize groups of phylotypes and lipids sharing similar correlation patterns. Analogous ‘second-order’ correlations have previously been applied for instance in the context of gene expression studies (Parmigiani et al., 2004; Lahti et al., 2013). A constant plaid model biclustering was used for systematic detection of significantly correlated bacterial and lipid groups on the correlation heatmap (Lazzeroni & Owen, 2002). To interpret the detected biclusters, statistical enrichment (over-representation) of the implicated bacteria was quantified. Moreover, enrichment analysis was carried out for lipids containing even or odd number of carbon atoms, as well as the enrichment of lipids with zero, one or more double bonds. The enrichment analyses were carried out with Fisher’s exact test (Rivals et al., 2007). All analyses were performed within the R statistical environment (R Development Core Team, 2010).

## Results

### Impact of the *L. rhamnosus* GG intervention in the intestinal microbiota

This study characterized the impact of *L. rhamnosus* GG intervention on the stability and composition of the intestinal microbiota. The subjects in the probiotic group consumed daily approximately 10^10^ (10.2log_10_) colony forming units (cfu) of *L. rhamnosus* GG. The compliance was verified with the quantification of *L. rhamnosus* GG in the feces with strain-specific qPCR (Kekkonen et al., 2008b). The average excretion of *L. rhamnosus* GG in the probiotic group was more than 1000-fold higher than that in the placebo group (8.52; sd 0.73log_10_ versus 5.20; sd 1.09log_10_ genome copies per gram of feces, respectively).

**Figure 2:**
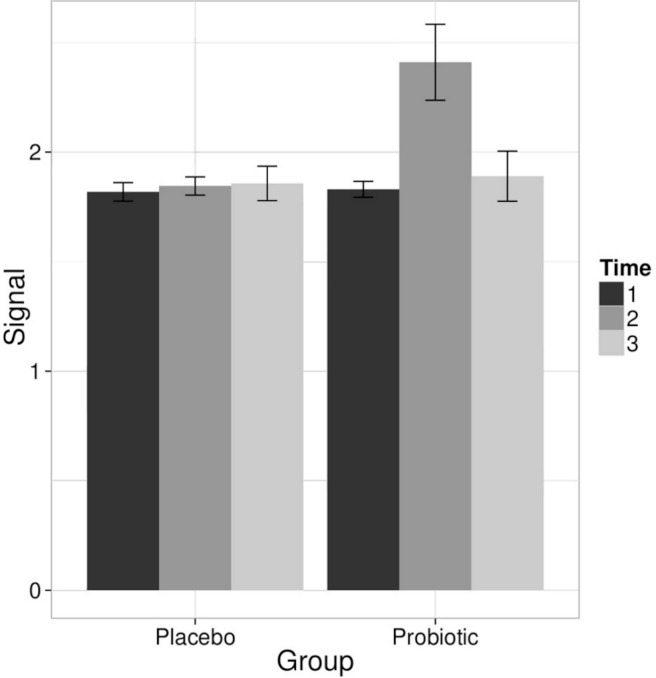
Intervention effects on the abundance of *L. rhamnosus*. Mean abundance of *L. rhamnosus* among the study subjects before, during and after the probiotic intervention (the time points 1–3, respectively) quantified by the HITChip hybridization signal. The error bars denote the Gaussian 95% confidence limits based on standard deviation of the mean.

The stability of the microbiota during the trial was quantified by the similarity of the microbiota profiles between the three time points with Pearson correlation (Table 1). The average intra-individual correlations were high; 0.94–0.95 (sd 0.02–0.03). No significant difference in the temporal stability of the microbiota between the probiotic and placebo groups was observed, indicating that the probiotic intervention did not alter the overall microbial stability. Principal Component Analysis (PCA) visualization of the relationships between the intestinal microbiota profiles (Fig. 2A) and hierarchical clustering (data not shown) further supported the conclusion that there were no systematic differences in the microbiota between the intervention groups. The average inter-individual microbiota correlation was 0.76–0.78 with no significant differences between the probiotic and placebo groups (Table 1). The intra-individual microbiota correlations (*r* = 0.94–0.95) were notably higher than the inter-individual correlations (*r* = 0.76–0.78), stressing the subject-specificity of the microbiota.

**Table 1:** Stability of microbiota and lipid profiles in the probiotic and placebo groups. We determined the similarity (expressed as Pearson’s correlation) both within and between the time points (TP) for the microbiota and lipid profiles by the average scatter *r* of the profiles. Lipid data is available for the first two time points (TP1 and TP2, three weeks before the intervention and during the intervention, respectively), and not available (−) for the third time point (TP3) measured three weeks after the intervention.

	Between subjects			Within subjects	
**Microbiota**	TP1	TP2	TP3	TP1 vs TP2	TP3 vs TP2
Probiotic	0.78	0.78	0.78	0.94	0.95
Placebo	0.76	0.77	0.77	0.94	0.95
					
**Lipids**					
Probiotic	0.90	0.89	–	0.92	–
Placebo	0.91	0.89	–	0.93	–

Linear models were used to quantify the effects of the *L. rhamnosus* GG intervention on individual taxa using genus- and phylotype-level microarray data. A specific and transient increase of bacteria related to *L. rhamnosus* was detected in the probiotic group immediately after the intervention (Fig. 2). There were no intervention-related effects in the other lactobacilli, Bifidobacteria or any other taxa either in the probiotic or placebo group; however substantial individual variation was evident.

To complement the HITChip microarray analysis, we also determined the absolute counts of total bacteria, methanogenic Archaea, *Lactobacillus* group and *Bifidobacterium* spp. using real-time PCR. The ingestion of *L. rhamnosus* GG was reflected in the total lactobacilli that showed a highly significant increase in the probiotic group after the intervention, returning to baseline levels in the follow up (*q* < 0.05 in both comparisons). No other significant changes were observed in the amount of targeted microbes neither in the probiotic or placebo group. We detected substantial inter- and intra-individual variation in the methanogenic Archaea but that was independent of the time point or the treatment group (data not shown). These observations support the conclusion that the *L. rhamnosus* GG intervention did not change the overall microbiota composition.

### High-throughput profiling of serum lipids

Global profiling of the serum lipids was performed using the UPLC-MS platform to assess the serum lipid profiles at the molecular species level (Kekkonen et al., 2008a). In total, 407 lipid species from 11 different classes were identified. The lipid profiles were dominated with triglycerides (TG; 37%), phosphatidylcholines (PC; 25%) and phosphatidyletanolamines (PE; 13%) while phosphatidic acids (PA), phosphatidylglycerols (PG), sphingomyelins (SM), cholesteryl esters (ChoE), lysophosphatidylcholines (lysoPC), phosphatidylserines (PS), ceramides (Cer) and lysophosphatidylethanolamines (lysoPE) each contributed with 7% to 0.3% to the total lipid pool.

The stability of the lipid profiles was quantified based on the same correlation analysis approach than in the HITChip microbiota profiling analysis. No significant differences in the lipid profile stability were observed between the treatment groups (average intra-individual *r* = 0.92 and *r* = 0.93 for the probiotic and placebo groups, respectively; Table 1). In contrast to microbiota profiles, the serum lipid profiles were remarkably similar not only within the subjects but also between the subjects (average inter-individual *r* = 0.89–0.91; Table 1). No significant differences were seen between the probiotic and placebo groups, or between the time points. The stability of the lipid and microbiota profiles showed a weak positive correlation (*r* = 0.27) across the subjects independent of a treatment group. The global lipid profiles did not separate according to the treatment group in PCA visualization (Fig. 2B), and no statistically significant lipid alterations were detected between the time points neither in the probiotic or placebo group (data not shown; Kekkonen et al., 2008b). The fold-changes for each lipid species between the two time points followed normal distribution in both intervention groups (data not shown), which further supports the conclusion that the intervention did not induce systematic alterations on the lipid profiles.

### Associations between the intestinal microbiota and serum lipids

In addition to studying the effects of probiotic *L. rhamnosus* GG intervention in the intestinal microbiota, we investigated the co-variation of the microbiota and serum lipid profiles. As discussed above, our results (Table 1, Fig. 2) conclusively show that the probiotic intervention did not affect the lipid profiles or the microbiota beyond the *L. rhamnosus* that was ingested. Hence, the subjects from probiotic and placebo groups were pooled to increase the sample size and statistical power in exploring the associations between the intestinal microbiota and serum lipids.

**Figure 3:**
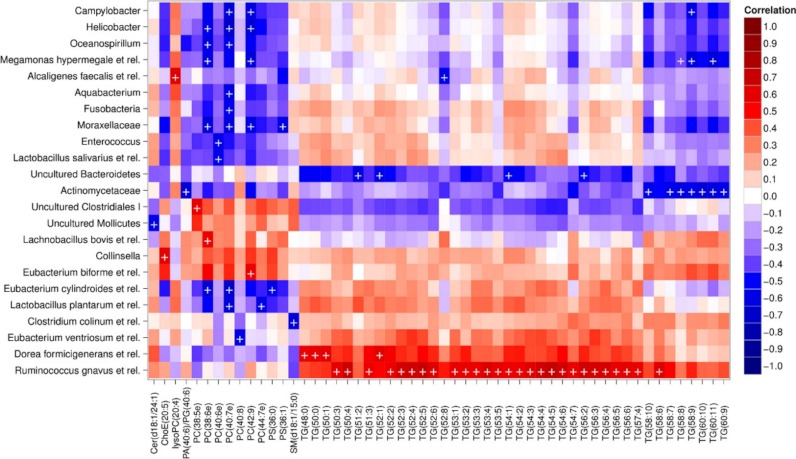
Correlations between intestinal genus-level phylogenetic groups and serum lipids. The correlations between the intestinal bacteria and serum lipids are indicated by colors (red: positive; blue: negative). The significant correlations (*q* < 0.05) are indicated by ‘ + ’; only lipids and bacteria with at least one significant correlation are shown. Hierarchical clustering of the rows and columns highlights groups of significantly correlated bacteria and lipids. Lipids have been named according to Lipid Maps (http://www.lipidmaps.org) with the following abbreviations: Cer: ceramide; ChoE: cholesteryl ester; lysoPC: lysophosphatidylcholine; PA: phosphatidic acid; PG: phosphatidylglycerol; PC: phosphatidylcholine; PS: phosphatidylserine; SM: sphingomyelin; TG: triglyceride. Where the fatty acid composition could not be determined, the total number of carbons and double bonds is indicated. The first number indicates the amount of carbon atoms in the fatty acid molecule, followed by the number of double bonds. For further details, see the Methods section.

Heatmap visualization of the microbiota–lipid correlations across the 22 subjects from 2 time points revealed intestinal bacteria and serum lipids with significantly correlated abundance patterns (Fig. 3). In total, 86 bacterial group-lipid pairs with notable correlations ( ± 0.5 or higher) were identified at *q* < 0.05 significance level (Fig. 3). Among the significant correlations, 23 of the 131 genus-level taxa detectable by the HITChip were represented (Supplemental Table S3). Most of these taxa belonged to Proteobacteria (6) and Firmicutes within Clostridium cluster XIVa (5) and Bacilli (3). From the lipid side, the vast majority of the significant correlations were attributable to the most dominant lipids, TGs (62%) and PCs (30%). When analyzed at the genus-level, the significant correlations were strongly dominated by the positive correlations between bacteria related to *Ruminococcus gnavus* and different TG species (31 of 40 significant positive correlations, average *r* = 0.61; Fig. 3A). Detailed analysis at the phylotype-level data indicated that within the *R. gnavus* group, two uncultured phylotypes, uncultured human gut bacterium JW1G3 and JW1H4a, dominated the correlations while the type species *R. gnavus* or related *R. torques* did not correlate significantly with TG or any other lipid species. Other representatives of the *Clostridium* cluster XIVa included bacteria related to *Dorea formicigenerans* that also correlated positively with four TG species (Fig. 3; average *r* = 0.58). The remaining TG correlations were negative and involved phylogenetically diverse bacteria (Supplemental Table S3). Also PCs correlated significantly with numerous taxa, most of which correlated negatively with ester- or ether linked PCs. Interestingly, a set of Proteobacteria (*Campylobacter*, *Helicobacter* and *Moraxellaceae*) with low signal intensities had a clear negative correlation with PC. Similarly, two *Lactobacillus* species (*L. rhamnosus* that was ingested during the trial and *L. salivarius*) together with another group of *Bacillus*, *Enterococcus* spp., also correlated negatively with PC while few Firmicutes exhibited a positive correlation with this lipid species. Among the less abundant lipids (<10% of the total lipid pool), a positive correlation was detected between cholesteryl ester and *Collinsella* (*r* = 0.59; Supplemental Table S3, Fig. 5A). On the phylotype level, the correlation was mainly attributable to an uncultured bacterium clone Eldhufec074 within genus *Collinsella*. Another group of Actinobacteria, namely *Actinomycetaceae*, showed a significant negative correlation to TG and PA.

Hierarchical clustering and heatmap visualization of the significantly correlated lipid–bacteria pairs revealed groups of lipids and bacteria sharing similar correlation patterns. A constant plaid model biclustering approach was applied to detect coherent groups of significantly correlating microbe–lipid pairs (see Methods). The analysis revealed three major clusters of significantly correlated lipid–microbe pairs (Supplemental Table S2). One of the observed biclusters highlights the above discussed positive association between bacteria related to *R. gnavus* and TGs. Another TG cluster, containing highly unsaturated long-chain fatty acids, consisted of negatively correlating bacteria related to *Megamonas hypermegale* (family *Veillonellaceae*, Clostridium cluster IX) or belonging to the *Actinomycetaceae*. The third cluster contained a set of Proteobacteria (*Helicobacter, Oceanospirillum and Moraxellaceae spp.*) and bacteria related to *Eubacterium cylindroides* within the family *Erysipelotrichaceae*, which all correlated negatively with ether lipids.

To explore the nature of the identified lipid–microbe pairs, we quantified the enrichment of specific, functionally relevant lipid categories in the biclusters. In particular, we investigated the enrichment of lipids with even and odd number of carbon atoms and the degree of saturation (0, 1, or more double bonds). The origin of lipid can be inferred from its chemical composition: Polyunsaturated fatty acids (PUFAs; more than one double bond) and lipids that have uneven number of carbon atoms originate from plants, bacteria or marine organism as humans cannot synthesize them. Significant enrichment of long acyl chain (*p* < 0.05; Fisher exact test) was detected among the TG lipids that significantly correlated with bacteria related to *R. gnavus* (Supplemental Fig. S1; Supplemental Table S2). Such a positive association suggests a role for these *R. gnavus-*related bacteria in the absorption of dietary lipids. In support of this interpretation, TGs containing fatty acids with odd number of carbons were also relatively common in this group (*p* = 0.1). In the other two biclusters, no significant enrichment of odd/even carbon count or saturation level was detected but within the bicluster 2 (Supplemental Table S2), *Actinomycetaceae* correlated exclusively with highly unsaturated PUFAs (Fig. 3).

### Associations between the intestinal microbiota and biochemically determined serum lipids

**Figure 4:**
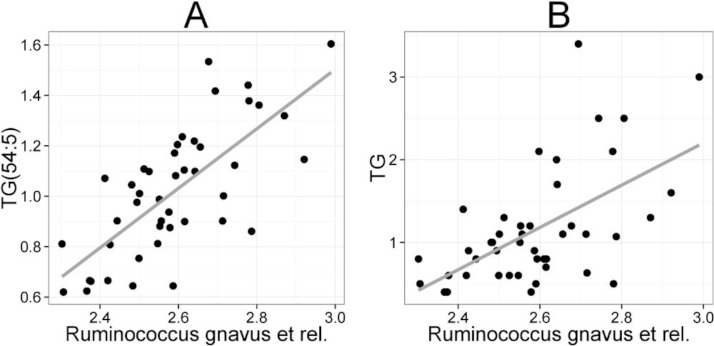
Association between *Ruminococcus gnavus et rel.* and serum triglyceride (TG) lipids. The relative amounts of *R. gnavus et rel.* were quantified by the HITChip analysis and the triglyceride concentration was determined based on two independent techniques: **A** the triglyceride TG(54:5) (see Fig. 3 for explanation) by mass spectrometry (*r* = 0.61); **B** triglyceride by an enzymatic assay (*r* = 0.60).

The mean values (SD) for the major serum lipids were total cholesterol 5.10 (1.02), LDL cholesterol 3.00 (1.21), HDL cholesterol 1.50 (0.33) and TG 1.20 (0.71) mmol/L (Kekkonen et al., 2008a). No significant changes were detected in the lipids of the probiotic or the placebo groups during the intervention (Kekkonen et al., 2008b). Hence, we analyzed the potential associations of the enzymatically determined major blood lipids with the intestinal microbiota (Table 2). A positive correlation between bacteria related to *R. gnavus* and TG was observed (*q* < 0.01; *r* = 0.60; Fig. 4B), corroborating the association identified within the global lipid analysis. In line with the spectroscopic lipid analysis, representatives of Bacteroidetes and Uncultured *Clostridiales* correlated negatively and other implicated Firmicutes positively with enzymatically determined TG (Table 2). Representatives of Proteobacteria and Actinobacteria that correlated negatively with TG in the lipid profiling data did not correlate significantly with enzymatically determined TG. Such inconsistency may partly arise from methodological reasons as the enzymatic assay captures not only TG but also diacylglyceride, monoacylglyceride and free glycerol, while lipid profiling captures TGs at the molecular level.

**Table 2:** Associations between genus-level bacterial groups and enzymatically determined lipids. Associations between the relative amounts of genus-level bacterial groups as determined by the HITChip analysis and the serum lipid concentrations are quantified with a biweight midcorrelation. Only significant positive and negative correlations are shown (*q* < 0.05; otherwise ‘−’). Abbreviations: Total cholesterol (TC), high-density lipoprotein (HDL) and low-density lipoprotein (LDL) cholesterol, triglyceride (TG). Correlations between genus-level bacterial groups and mass spectrometry-determined lipids are provided in Supplemental Table S3.

Phyla/Firmicute order	Genus-level taxon	TC	LDL	HDL	TG
Actinobacteria	*Collinsella*	0.56	0.57	–	–
Bacilli	*Aneurinibacillus*	–	–	−0.58	0.50
Bacteroidetes	*Bacteroides plebeius et rel.*	–	–	–	−0.47
Bacteroidetes	*Bacteroides vulgatus et rel.*	–	–	–	−0.47
Bacteroidetes	*Tannerella et rel.*	–	–	–	−0.45
Clostridium cluster XI	*Anaerovorax odorimutans et rel.*	–	–	−0.48	0.49
Clostridium cluster XIVa	*Clostridium nexile et rel.*	–	–	−0.45	–
Clostridium cluster XIVa	*Clostridium sphenoides et rel.*	–	–	–	0.46
Clostridium cluster XIVa	*Dorea formicigenerans et rel.*	–	–	−0.56	0.57
Clostridium cluster XIVa	*Eubacterium hallii et rel.*	–	–	–	0.47
Clostridium cluster XIVa	*Ruminococcus gnavus et rel.*	–	–	−0.46	0.60
Clostridium cluster XIVa	*Ruminococcus obeum et rel.*	–	–	−0.48	0.51
Clostridium cluster XV	*Anaerofustis*	–	–	−0.45	–
Clostridium cluster XVI	*Eubacterium biforme et rel.*	0.48	0.47	–	–
Clostridium cluster XVI	*Eubacterium cylindroides et rel.*	–	–	−0.45	–
Uncultured Clostridiales	Uncultured *Clostridiales I*	–	–	0.53	−0.45
Uncultured Clostridiales	Uncultured *Clostridiales II*	–	–	–	−0.54

**Figure 5:**
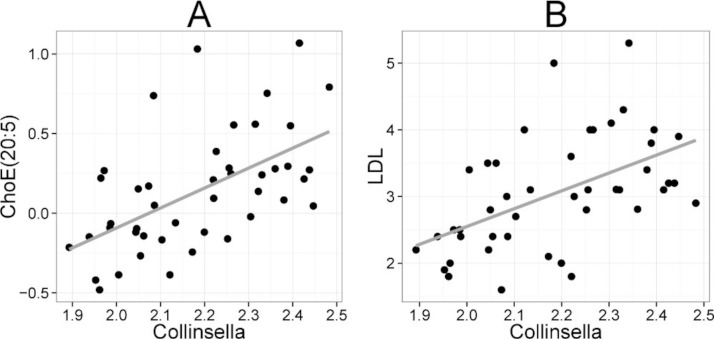
Association between *Collinsella spp.* and serum cholesterol. The relative amounts of *Collinsella spp.* were quantified by the HITChip analysis, and serum cholesterol levels were determined by two independent techniques: **A** Cholesterol ester ChoE(20:5) (see Fig. 3 for explanation) by mass spectrometry (*r* = 0.59); **B** low-density lipoprotein (LDL) cholesterol by enzymatic assay (*r* = 0.57).

*Collinsella* spp. and *Eubacterium biforme et rel.* showed statistically significant (*q* < 0.05) and positive correlations to enzymatically determined total and LDL cholesterol (Fig. 5B; Supplemental Table S2). No other significant correlations were identified for total or LDL cholesterol while HDL cholesterol correlated significantly with numerous taxa. Eight different Firmicutes including taxa related to *Ruminococcus obeum* and *D. formicigenerans* were found to correlate negatively with HDL, while Uncultured *Clostridiales* I was the only taxon showing positive correlation to HDL (Supplemental Table S2).

## Discussion

In the present study, we analyzed the effect of probiotic intake on the stability and composition of the intestinal microbiota and of the serum lipids, and the overall associations between the microbiota and lipid profiles. The microbiota was analyzed using the HITChip, a phylogenetic microarray, providing one of the first holistic and community-level microbiota assessments after a probiotic intervention. The data published so far is largely dominated with targeted microbiota analyses that have reported a generic increase of lactic acid bacteria after intake of individual lactobacilli strains (McNulty et al., 2011; Palaria, Johnson-Kanda & O’Sullivan, 2011; Satokari et al., 2001; Savard et al., 2011; Vaughan, Mollet & de Vos, 1999; Yamanoa et al., 2006). Our community-level analysis showed that the probiotic intake did not introduce any changes in the microbiota composition or stability except the specific increase of *L. rhamnosus* and total lactobacilli, which likely reflected the excretion of the ingested strain. Similarly, ingestion of a mixture of five different probiotic strains did not induce any significant changes in the microbiota composition neither in adults nor in simplified model community in mice (McNulty et al., 2011). Even when a combination of probiotic and prebiotic (synbiotic) food was consumed, two studies based on microbiota profiling and qPCR did not identify any differences in the microbiota composition between placebo and treatment besides the ingested strains (Palaria, Johnson-Kanda & O’Sullivan, 2011; Vitali et al., 2010).

We have shown previously using the same cohort that the ingestion of *L. rhamnosus* GG had apparent effects on the host immunology (Kekkonen et al., 2008b). Hence, our results does not support the hypothesis that probiotic bacteria would modulate the endogenous microbiota but rather points towards direct signaling to host or altered gene expression of the resident microbiota as a mode of probiotic action as recently proposed (McNulty et al., 2011).

Our study was carried out in healthy adults with seemingly well-established and balanced microbial communities. In subjects whose intestinal ecosystem is unbalanced e.g. due to pathogen overgrowth, gastrointestinal symptoms or recent intake of antibiotics, intake of probiotic bacteria may modulate the microbiota. Similarly, the developing microbiota of children is potentially more susceptible to environmental modulators including probiotics. Although substantial changes in infant microbiota following daily intake of *L. rhamnosus* GG have indeed been reported (Cox et al., 2010), these results must be interpreted with caution as the phylogenetic microarray used in that study provided signals for close to 50 phyla (Midgley et al., 2012) whereas the human intestinal ecosystem only contains a maximum of 10 phyla (Rajilić-Stojanović, Smidt & de Vos, 2007).

To the best of our knowledge, this cohort is the first addressing the potential impact of probiotics on global serum lipid profiles in adults. Daily intake of *L. rhamnosus* GG for three weeks did not introduce any consistent alterations in the serum lipids or in the biochemically determined cholesterol and triglyceride levels of the healthy, normolipidemic adults analyzed in this and previous study (Kekkonen et al., 2008a). Probiotic bacteria have been suggested as a potential non-drug treatment to lower serum cholesterol levels based on their *in vitro* described ability to deconjugate bile acids and directly assimilate cholesterol. A recent meta-analysis implies that probiotic intake can lower the total and LDL cholesterol (Guo et al., 2011), although numerous human studies have failed to observe any effects on serum lipids after probiotic intake (see e.g. Sadrzadeh-Yeganeh et al., 2010; Pereira & Gibson, 2002). One explanation for the controversy is that similarly to the strain-specificity of the immunomodulatory effects of probiotics (Kekkonen et al., 2008b), also their effects on lipid metabolism are likely to vary between different strains. In atopic infants the consumption of *L. rhamnosus* GG supplemented formulas for several months resulted in a reduced proportion of α-linolenic acid and of total n-3 PUFAs, leading to increased n-6 to n-3 PUFA ratio in sera of probiotic-fed infants (Kankaanpää et al., 2002). Decrease of n-3 fatty acids has negative health implication, underlining the need for more *in vivo* research to specify the bacterial strains and target population where probiotics can have beneficial effects on the host lipid metabolism.

Our data indicates that the variation of the intestinal microbiota is considerably higher across the individuals than the variation in serum lipid profiles, highlighting the tight homeostatic control of systemically circulating lipids. The intestinal microbiota is known to play an important role in the regulation of systemic lipid metabolism (Martin et al., 2007; Velagapudi et al., 2010). In this study, we identified several novel associations between the human intestinal commensals and serum lipids. From the lipid side, TGs (62%) and PCs (30%) dominated the significant lipid–microbe correlations. TGs are used for energy storage while PCs are abundant constituents of cell membranes. Both of these abundant lipid classes were affected upon colonization of germ-free mice (Velagapudi et al., 2010). The most prominent correlation was between TGs and uncultured phylotypes related to *R. gnavus* that belong to family *Ruminococcaceae* in Clostridium cluster XIVa. Two characterized species within the group, *R. gnavus* and *R. torques*, have been implicated in intestinal disorders as they appeared to be overrepresented both in IBS (Kassinen et al., 2007; Rajilić-Stojanović et al., 2011) and in IBD (Joossens et al., 2011; Prindiville, Cantrell & Wilson, 2004). The TG lipids that were positively associated with *R. gnavus*-related phylotypes were enriched with polyunsaturated and odd-chain fatty acids. Since the odd-chain fatty acids are not synthesized in the body, these positive associations suggest that the implicated bacteria facilitate the absorption of polyunsaturated dietary lipids. Moreover, it cannot be excluded that these bacteria are even involved in their biosynthesis as related *R. obeum* together with other Firmicutes are among the intestinal bacteria capable of producing isomers of conjugated linoleic acids (CLA; McIntosh et al., 2009).

Triglycerides carry different types of fatty acids, and accordingly not all TGs displayed the same pattern regarding microbial correlations. For example, bacteria related to *D. formicigenerans* showed positive association to saturated triglycerides with a small carbon number, i.e. including palmitic acid. These fatty acids are mainly produced *de novo* in the liver (Westerbacka et al., 2010; Kotronen et al., 2009). *D. formicigenerans* produces formate and acetate that are precursors of hepatic lipogenesis. Recent metaproteomic work verifies that the acetate kinases involved in acetate production are highly expressed in the intestinal ecosystem (Kolmeder et al., 2012), suggesting that several intestinal bacteria have potential to regulate lipogenesis. On the other hand, *Actinomycetaceae* were inversely associated with TGs containing highly unsaturated triglycerides with a high carbon number. Such TGs carry physiologically important fatty acids such as C22:6 (docosahexanoic acid) and C20:4 (arachidonic acid). Both are important cell membrane components, the former especially in visual and neural tissues, and the latter is involved in inflammatory signaling. While not much is known about the intestinal *Actinomycetaceae*, both commensal and pathogenic forms of these Actinobacteria are abundant in the oral cavity.

We identified multiple taxa correlating negatively with normal or ether linked forms of PC. Dietary PC was recently identified as compound that after conversion by the intestinal microbiota promotes heart disease (Wang et al., 2011). Unfortunately, that study did not address the identity of bacteria involved in the metabolism of dietary PC, but our data suggest that different Firmicutes, Proteobacteria and Fusobacteria may participate in this metabolic conversion (Fig. 3). Remarkably, the presence of serum ether PCs was negatively associated with various taxa, mainly with Gram-negative genera that include pathogens and commensals, such as *Helicobacter*, *Moraxellaceae* and *Campylobacter*. Among the ether lipids, plasmalogens, known as endogenous antioxidants (Wallner & Schmitz, 2011), are the most abundant. Their negative association with Proteobacteria could indicate that the implicated bacteria may induce oxidative stress in host cells, leading to depletion of plasmalogens. In support of this hypothesis, lipopolysaccharide (LPS), a ubiquitous and toxic surface component of Gram-negative bacteria, induces oxidative stress in mammalian cell cultures (Aly, Lightfoot & El-Shemy, 2010).

Finally, we identified positive correlation between the abundance of serum cholesterol and genus *Collinsella* (Actinobacteria, family *Coriobacteriacea*). Biochemical lipid analysis indicated that *Collinsella* spp. together with bacteria related to *E. biforme* correlated specifically with total cholesterol and LDL but not HDL. To our knowledge, our work provides the first *in vivo* evidence about the implication of *Coriobacteriacea* in human lipid metabolism. Our present findings are supported by the data generated with rodent models. In a hamster model, the proportion of *Coriobacteriaceae* showed high positive correlation with non-HDL plasma cholesterol levels and reacted to the intake of dietary lipids (Martinez et al., 2009). In a mouse model, a strong positive correlation between *Coriobacteriaceae* and hepatic triglycerides was identified (Claus et al., 2011), providing cumulative indication for the involvement of *Coriobacteriaceae* in mammalian lipid metabolism. While only one taxon (*Collinsella*) correlated statistically significantly with cholesterol in the lipid profiling data, numerous taxa showed significant correlation to enzymatically determined HDL cholesterol. This may arise from technical differences, as the lipid profiling does not quantify free cholesterol but its derivative cholesteryl ester, where cholesterol is esterified to long-chain fatty acids. All taxa that were associated with both HDL cholesterol and TG had opposite correlation with these lipids (Supplemental Table S2), in accordance with the fact that serum TG and HDL show strong inverse relationship, and their ratio is used as an atherogenic index that predicts cardiovascular risk (Jeppesen et al., 1997). Hence, identification of bacteria that potentially affect the athreogenic index may provide important clinical implications for dyslipidemic individuals.

While good correspondence between the HITChip and pyrosequencing studies have been previously demonstrated, the main advantage of the HITChip microarray compared to the pyrosequencing studies is that the microarray technology provides very standardized and cost-efficient tools for deep and reproducible analysis of intestinal microbiota including phylotypes that are only present in low concentrations (Claesson et al., 2009; Salonen et al., 2012). We expect that our major findings, the associations between specific lipid species and bacteria related to *Collinsella* or *R. gnavus* could be detected also in a standard pyrosequencing study due to the relatively high abundance of these organisms. However, the associations involving less abundant taxa (Bacilli, Proteobacteria, *Actinomycetaeae*) would likely have been missed with conventional sequencing depth.

In summary, quantitative analysis of high-throughput profiling data identified several significant correlations between the intestinal microbiota and serum lipids. These results partly confirm and extend previous observations in animal studies, and provide hypotheses for follow-up studies in humans. It is important to note, however, that the present analysis is based on a Finnish cohort and only two time points, and does not consider causal relationships between these variables. Therefore it cannot be excluded that some of the observed correlations may be associated with the diet or other confounding variables that simultaneously affect both lipid and microbiota profiles. However, as all subjects ate their habitual diets, individual food components are not likely to cause systematic bias to the observed lipid–microbe correlations, which is further supported by the observation of distinct groups of correlated lipid–microbe pairs in the bicluster analysis. It can also be ruled out that the intervention would have caused the correlations as control measurements from the baseline were included, the ingested probiotics *L. rhamnosus* GG did not correlate significantly with any serum lipid, and the drink did not otherwise alter the microbiota. Confirmation of the findings in an independent cohort and a more thorough longitudinal analysis will be necessary to assess the effect of external variables on the microbiota–lipid correlations and their potential causal associations. None of the implicated bacterial taxa are functionally characterized, and thus the potential mechanisms of how human intestinal bacteria relate to the host lipid metabolism are currently unknown. Among the potential mechanisms, bacterial modification of the bile acid pool is by far the best characterized. *Collinsella* spp., *R. gnavus* as well as the genus *Eubacterium* are among the intestinal bacteria capable of deconjugating bile acids (Lepercq et al., 2004). Bile acids are cholesterol-derived detergents that play a central role in the absorption of fat in the intestine but also in signaling with systemic endocrine functions (Swann et al., 2011). It has been known for long that intestinal bacteria can chemically modify bile acids but recent work suggest that the microbiota may also control their production and degradation (Antunes et al., 2011). In mice the intestinal bacteria profoundly affect the emulsification, absorption and transport of dietary fat as well as their storage and peroxidation through the metabolic and signaling properties of bile acids (Martin et al., 2007).

## Conclusion

Our data supports the concept that the overall lipid content in human serum is a composite of host and microbial metabolic activity, and the intestinal commensals are implicated in the metabolism of various lipid species that human body utilizes for membranes, energy storage and signaling. Considering that a single gene in an intestinal bacterium could alter host fatty acid composition (Rosberg-Cody et al., 2011), we can only envisage the metabolic capacity and the functional consequences from the million genes in the intestinal microbiome. As epidemiological data do not support a link between dietary cholesterol and serum cholesterol levels (Lecerf & de Lorgeril, 2011), the role of genetic factors in the individual variability of cholesterol levels is evident. Identification of the bacteria and their mechanisms that regulate host fatty acid and lipid metabolism have considerable potential for clinical implications due to the profound role of these molecules for instance in cardiovascular disease and regulation of inflammatory cytokine signaling (Calder, 2011). Future trials involving controlled diet and dyslipidemic individuals will provide further insights on the role of intestinal microbes on human lipid metabolism.

## Supplemental Information

10.7717/peerj.32/supp-1 Supplemental Fig. S1 Categorization of lipids that correlate significantly with intestinal bacteria. Significant correlations (*q* < 0.05, correlation +/−0.5 or higher) between lipids and genus-level groups of bacteria are organized according to the A) number of double bonds and B) number of carbons in acyl chain. The direction of correlation is visualized with colors (red: positive; blue: negative correlation). The degree of saturation (A) and carbon count (B) increase from left to right. SAFA: saturated fatty acid; MUFA: monounsaturated fatty acid; PUFA: polyunsaturated fatty acid.

10.7717/peerj.32/supp-2 Supplemental Information 1 Supplementary Data. The lipidomics and HITChip microbiota profiling data sets.

10.7717/peerj.32/supp-3 Supplemental Information 2 Supplementary Document. Supplementary Figures and Table.

10.7717/peerj.32/supp-4 Supplemental Table S1 Supplementary Document. Background information for the study subjects: subject ID (ID), intervention group (placebo or probiotics (*L. rhamnosus* GG)), gender, age, and body-mass index (BMI).

10.7717/peerj.32/supp-5 Supplemental Table S2 Supplementary Document. Clusters of significantly correlated lipid–phylotype pairs detected by bicluster analysis.

10.7717/peerj.32/supp-6 Supplemental Table S3 Summary of statistically significant lipid–bacteria correlations based on spectrometrically determined lipids. Lipids have been named according to Lipid Maps (http://www.lipidmaps.org) with the following abbreviations: Cer: ceramide; ChoE: cholesteryl ester; lysoPC: lysophosphatidylcholine; PA: phosphatidic acid; PG: phosphatidylglycerol; PC: phosphatidylcholine; PS: phosphatidylserine; SM: sphingomyelin; TG: triglyceride. Where the fatty acid composition could not be determined, the total number of carbons and double bonds is indicated. The first number indicates the amount of carbon atoms in the fatty acid molecule, followed by the number of double bonds.

## Additional Information and Declarations

### Microarray Data Deposition

The following information was supplied regarding the deposition of microarray data: The microarray data presented in the manuscript are phylogenetic microarray data for which the standards of commercial microarrays are not as such applicable. Therefore we have attached the data as Supplemental material of this manuscript, and details of the samples, experimental design, and study protocol are provided in the main text. Prior to this manuscript, this particular phylogenetic microarray, the HITChip, has been utilized in various publications, including: Claesson et al. PLoS ONE. 2009; 4(8):e6669, Biagi et al. PLoS ONE. 2010 May 17; 5(5):e10667 and Jalanka-Tuovinen et al. PLoS ONE. 2011; 6(7):e23035.

### Clinical Trial Registration

The following information was supplied regarding Clinical Trial registration: The trial and its protocol have been approved by the Ethics Committee of the Hospital District of Helsinki and Uusimaa (Ethical protocol no HUS 357/E0/05).
