# Supplementary figures and images for: Associations between the human intestinal microbiota, Lactobacillus rhamnosus GG and serum lipids indicated by integrated analysis of high-throughput profiling data
Source: PeerJ. 2013 Feb 26;1:e32. doi: 10.7717/peerj.32 (PMC3628737; doi:10.7717/peerj.32)

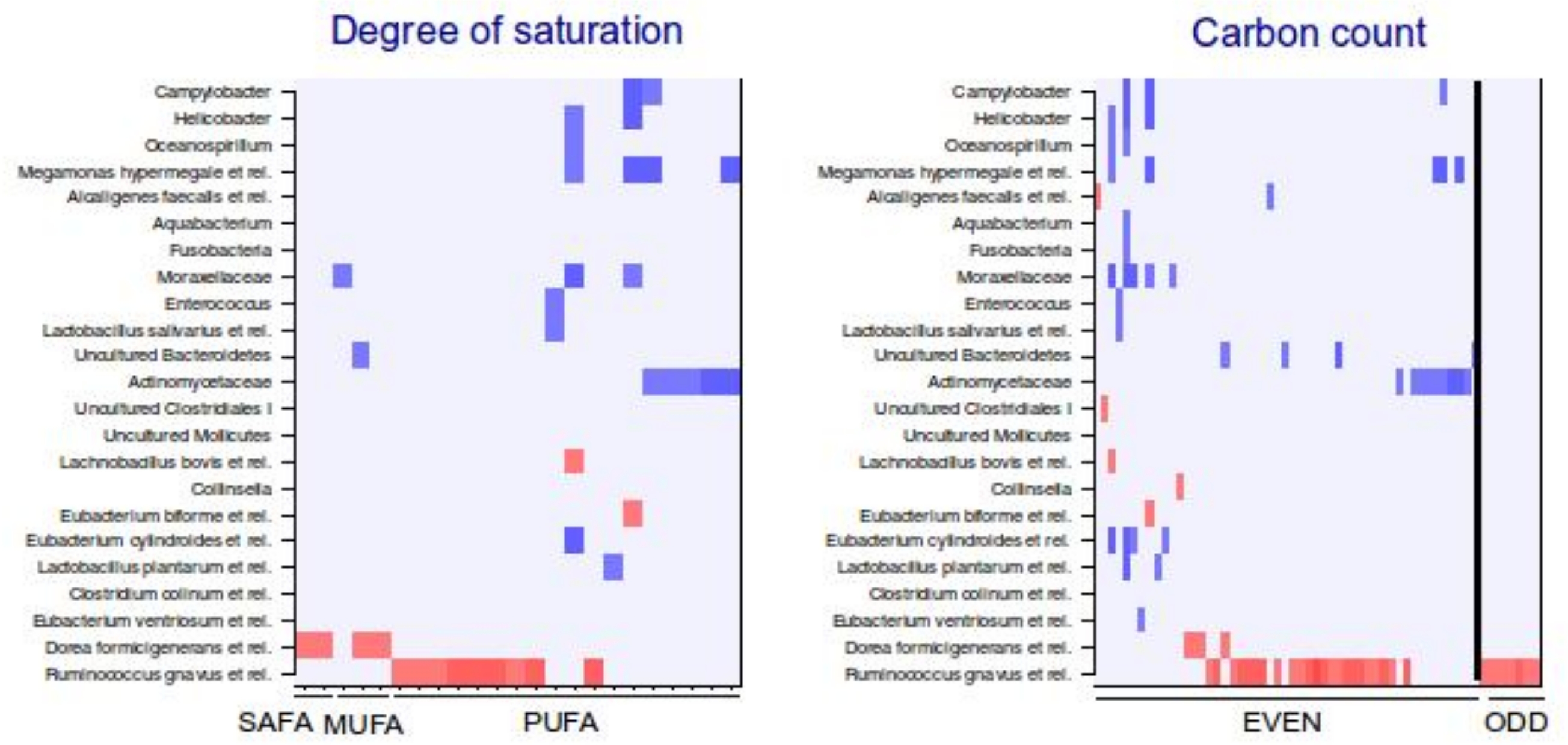

Supplement: Supplemental Fig. S1 — Significant correlations (q < 0.05, correlation +/−0.5 or higher) between lipids and genus-level groups of bacteria are organized according to the A) number of double bonds and B) number of carbons in acyl chain. The direction of correlation is visualized with colors (red: positive; blue: negative correlation). The degree of saturation (A) and carbon count (B) increase from left to right. SAFA: saturated fatty acid; MUFA: monounsaturated fatty acid; PUFA: polyunsaturated fatty acid. [file peerj-01-32-s001.jpg]
