# Supplementary material for: Associations between the human intestinal microbiota, Lactobacillus rhamnosus GG and serum lipids indicated by integrated analysis of high-throughput profiling data
Source: PeerJ. 2013 Feb 26;1:e32. doi: 10.7717/peerj.32 (PMC3628737; doi:10.7717/peerj.32)
Supplement: Supplemental Information 2 — Supplementary Figures and Table. [file peerj-01-32-s003.pdf]

Supplementary material

Associations between the human intestinal microbiota, *Lactobacillus rhamnosus* GG and serum lipids indicated by integrated analysis of high-throughput profiling data

Leo Lahti, Anne Salonen, Riina A. Kekkonen, Jarkko Salojärvi, Jonna Jalanka-Tuovinen, Airi Palva, Matej Orešič, and Willem M. de Vos

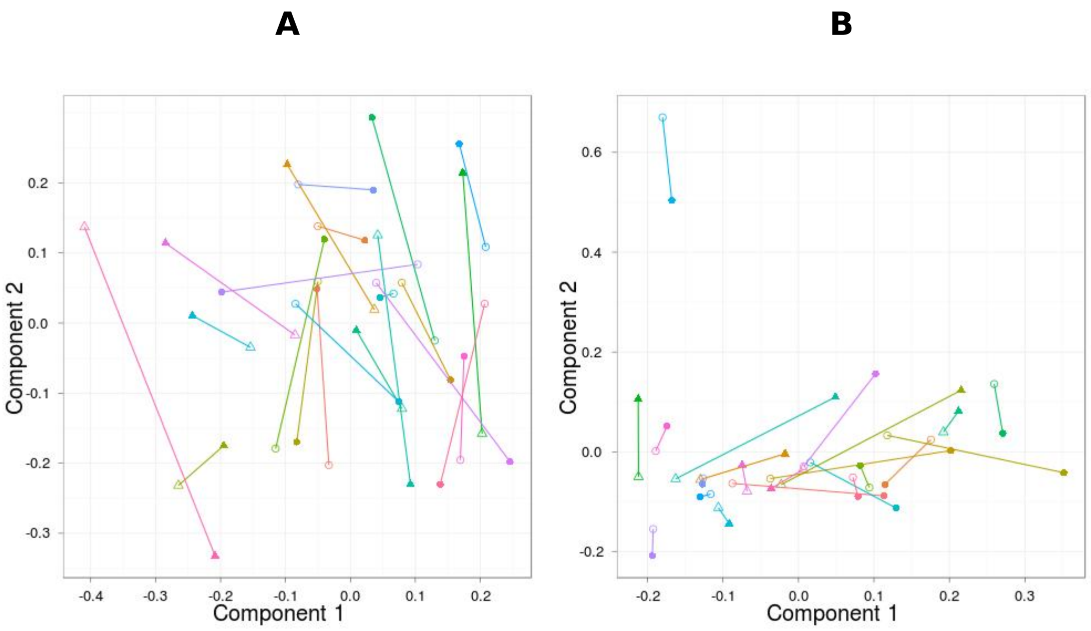

Figure S1 Principal component visualization of the intestinal microbiota and serum lipidomic profiles during probiotic intervention. Sparse Principal Component Analysis (PCA) of **A** microbiota **B** lipid profiles. The pre- and post-intervention samples are indicated by open and filled symbols, respectively. The triangles (probiotics) and circles (placebo) indicate the treatment group. The subjects are indicated by colors, and samples from the same subject are connected.

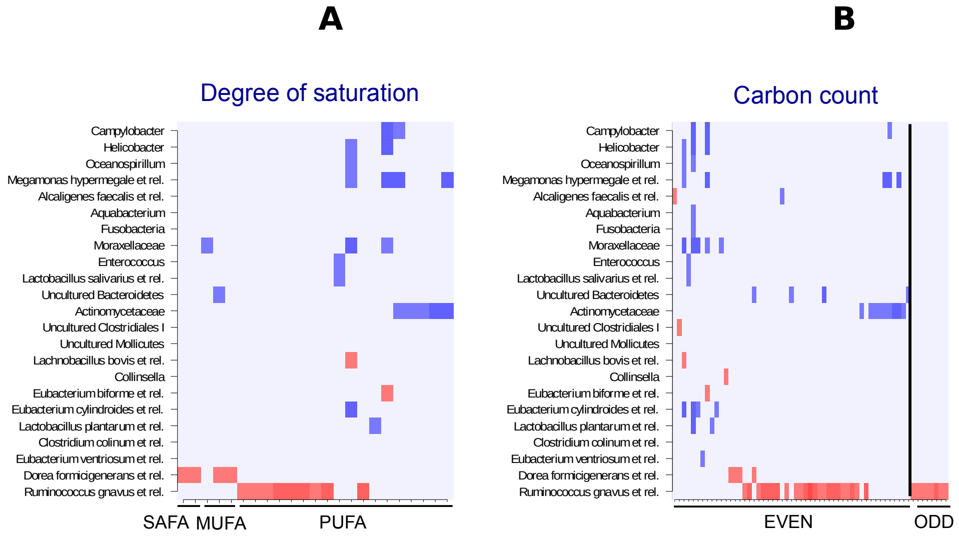

Figure S2 Categorization of lipids that correlate significantly with intestinal bacteria. Significant correlations ($q<0.05$, correlation +/- 0.5 or higher) between lipids and genus-level groups of bacteria are organized according to the **A)** number of double bonds and **B)** number of carbons in acyl chain. The direction of correlation is visualized with colors (red: positive; blue: negative correlation). The degree of saturation (A) and carbon count (B) increase from left to right. SAFA: saturated fatty acid; MUFA: monounsaturated fatty acid; PUFA: polyunsaturated fatty acid.

Table S1 Background information for the study subjects: subject ID (ID), intervention group (placebo or probiotics (*L. rhamnosus* GG)), gender, age, and body-mass index (BMI).

ID	Group	Gender	Age (y)	BMI
104	Placebo	Female	52	23.5
106	Placebo	Female	45	21.7
107	Probiotic	Female	41	21.6
112	Placebo	Male	52	26.1
113	Placebo	Female	55	28.4
116	Probiotic	Female	54	30.4
119	Placebo	Female	23	27.5
120	Probiotic	Female	54	23.4
122	Placebo	Male	26	21.3
123	Probiotic	Female	38	22.8
124	Probiotic	Female	48	21.9
127	Placebo	Female	28	24.3
130	Probiotic	Male	51	26.2
134	Placebo	Female	41	23.1
135	Probiotic	Female	46	18.4
136	Placebo	Female	46	20.3
137	Placebo	Female	27	24.8
139	Placebo	Female	34	26.1
145	Placebo	Male	43	24.7
152	Placebo	Male	37	22.4
155	Probiotic	Male	47	25.6
158	Placebo	Female	53	20.7
162	Probiotic	Male	50	28.1
163	Placebo	Female	32	21.5
164	Probiotic	Female	37	22.3

Table S2 Clusters of significantly correlated lipid-phylotype pairs detected by bicluster analysis.

Bicluster 1:
- Genus-level groups: *Ruminococcus gnavus et rel.*
- Lipids: *Triglycerides (TG) 50:3-4; 51:3; 52:2-6; 53:1-3,5; 54:1-7; 56:2-6; 57:4; 58:6*

Bicluster 2:
- Genus-level groups: *Megamonas hypermegale et rel.; Actinomycetaceae*
- Lipids: *Triglycerides (TG): 58:8-9; 60:11*

Bicluster 3:
- Genus-level groups: *Helicobacter; Oceanospirillum; Moraxellaceae; Eubacterium cylindroides et rel.*
- Lipids: *Phosphatidylcholine (PC): 40:7e; 38:6e*

Table S3 Summary of statistically significant lipid-bacteria correlations based on spectrometrically determined lipids. Lipids have been named according to Lipid Maps (http://www.lipidmaps.org) with the following abbreviations: Cer: ceramide; ChoE: cholesteryl ester; lysoPC: lysophosphatidylcholine; PA: phosphatidic acid; PG: phosphatidylglycerol; PC: phosphatidylcholine; PS: phosphatidylserine; SM: sphingomyelin; TG: triglyceride. Where the fatty acid composition could not be determined, the total number of carbons and double bonds is indicated. The first number indicates the amount of carbon atoms in the fatty acid molecule, followed by the number of double bonds.

**Phyla/Firmicute order**	**Genus-level taxon**	**PC**	**TG**	**Cer**	**ChoE**	**lysoPC**	**PA**	**PS**	**SM**
Actinobacteria	*Actinomycetaceae*	-	-0.58	-	-	-	-0.61	-	-
Actinobacteria	*Collinsella*	-	-	-	0.59	-	-	-	-
Bacilli	*Lactobacillus plantarum et rel.*	-0.60	-	-	-	-	-	-	-
Bacilli	*Enterococcus*	-0.57	-	-	-	-	-	-	-
Bacilli	*Lactobacillus salivarius et rel.*	-0.56	-	-	-	-	-	-	-
Bacteroidetes	*Uncultured Bacteroidetes*	-	-0.59	-	-	-	-	-	-
Clostridium cluster IX	*Megamonas hypermegale et rel.*	-0.60	-0.62	-	-	-	-	-	-
Clostridium cluster XIVa	*Ruminococcus gnavus et rel.*	-	0.61	-	-	-	-	-	-
Clostridium cluster XIVa	*Dorea formicigenerans et rel.*	-	0.58	-	-	-	-	-	-
Clostridium cluster XIVa	*Clostridium colinum et rel.*	-	-	-	-	-	-	-	-0.60
Clostridium cluster XIVa	*Eubacterium ventriosum et rel.*	-0.58	-	-	-	-	-	-	-
Clostridium cluster XIVa	*Lachnobacillus bovis et rel.*	0.56	-	-	-	-	-	-	-
Clostridium cluster XVI	*Eubacterium cylindroides et rel.*	-0.62	-	-	-	-	-	-0.57	-
Clostridium cluster XVI	*Eubacterium biforme et rel.*	0.56	-	-	-	-	-	-	-
Fusobacteria	*Fusobacteria*	-0.57	-	-	-	-	-	-	-
Proteobacteria	*Moraxellaceae*	-0.62	-	-	-	-	-	-0.58	-
Proteobacteria	*Campylobacter*	-0.62	-0.58	-	-	-	-	-	-
Proteobacteria	*Helicobacter*	-0.63	-	-	-	-	-	-	-
Proteobacteria	*Alcaligenes faecalis et rel.*	-	-0.56	-	-	0.59	-	-	-
Proteobacteria	*Oceanospirillum*	-0.59	-	-	-	-	-	-	-
Proteobacteria	*Aquabacterium*	-0.60	-	-	-	-	-	-	-
Uncultured Clostridiales	Uncultured *Clostridiales I*	0.56	-	-	-	-	-	-	-
Uncultured Mollicutes	Uncultured *Mollicutes*	-	-	-0.57	-	-	-	-	-
