# Supplementary material for: Associations between the human intestinal microbiota, Lactobacillus rhamnosus GG and serum lipids indicated by integrated analysis of high-throughput profiling data
Source: PeerJ. 2013 Feb 26;1:e32. doi: 10.7717/peerj.32 (PMC3628737; doi:10.7717/peerj.32)
Supplement: Supplementary file 6 [file peerj-01-32-s004.odt]

| ID | Group | Gender | Age (y) | BMI |
| --- | --- | --- | --- | --- |
| 104 | Placebo | Female | 52 | 23.5 |
| 106 | Placebo | Female | 45 | 21.7 |
| 107 | Probiotic | Female | 41 | 21.6 |
| 112 | Placebo | Male | 52 | 26.1 |
| 113 | Placebo | Female | 55 | 28.4 |
| 116 | Probiotic | Female | 54 | 30.4 |
| 119 | Placebo | Female | 23 | 27.5 |
| 120 | Probiotic | Female | 54 | 23.4 |
| 122 | Placebo | Male | 26 | 21.3 |
| 123 | Probiotic | Female | 38 | 22.8 |
| 124 | Probiotic | Female | 48 | 21.9 |
| 127 | Placebo | Female | 28 | 24.3 |
| 130 | Probiotic | Male | 51 | 26.2 |
| 134 | Placebo | Female | 41 | 23.1 |
| 135 | Probiotic | Female | 46 | 18.4 |
| 136 | Placebo | Female | 46 | 20.3 |
| 137 | Placebo | Female | 27 | 24.8 |
| 139 | Placebo | Female | 34 | 26.1 |
| 145 | Placebo | Male | 43 | 24.7 |
| 152 | Placebo | Male | 37 | 22.4 |
| 155 | Probiotic | Male | 47 | 25.6 |
| 158 | Placebo | Female | 53 | 20.7 |
| 162 | Probiotic | Male | 50 | 28.1 |
| 163 | Placebo | Female | 32 | 21.5 |
| 164 | Probiotic | Female | 37 | 22.3 |
