# Supplementary material for: Associations between the human intestinal microbiota, Lactobacillus rhamnosus GG and serum lipids indicated by integrated analysis of high-throughput profiling data
Source: PeerJ. 2013 Feb 26;1:e32. doi: 10.7717/peerj.32 (PMC3628737; doi:10.7717/peerj.32)
Supplement: Supplementary file 7 [file peerj-01-32-s005.odt]

Bicluster 1:

- Genus-level groups: Ruminococcus gnavus et rel.

- Lipids: Triglycerides (TG) 50:3-4; 51:3; 52:2-6; 53:1-3,5; 54:1-7; 56:2-6; 57:4; 58:6

Bicluster 2:

- Genus-level groups: Megamonas hypermegale et rel.; Actinomycetaceae

- Lipids: Triglycerides (TG): 58:8-9; 60:11

Bicluster 3:

- Genus-level groups: Helicobacter; Oceanospirillum; Moraxellaceae; Eubacterium cylindroides et rel.

- Lipids: Phosphatidylcholine (PC): 40:7e; 38:6e
