# Supplementary material for: Associations between the human intestinal microbiota, Lactobacillus rhamnosus GG and serum lipids indicated by integrated analysis of high-throughput profiling data
Source: PeerJ. 2013 Feb 26;1:e32. doi: 10.7717/peerj.32 (PMC3628737; doi:10.7717/peerj.32)
Supplement: Supplementary file 8 [file peerj-01-32-s006.odt]

OLE-object
